# Epidermal growth factor receptor (EGFR) inhibitor induced purpuric drug eruption: Three case reports

**DOI:** 10.1097/MD.0000000000018112

**Published:** 2019-11-22

**Authors:** Szu-Yun Fang, Chieh-Shan Wu, Yi-Shan Liu, Kai-Che Wei

**Affiliations:** a Department of Dermatology, Kaohsiung Veterans General Hospital; b Department of Dermatology, E-Da Hospital & I-Shou University, Graduate Institute of Science Education and Environmental Education, National Kaohsiung Normal University, Kaohsiung, Taiwan.

**Keywords:** acneiform eruption, epidermal growth factor receptor, epidermal growth factor receptor inhibitor, lung cancer, purpuric drug eruption

## Abstract

**Introduction:**

Purpuric drug eruption (PDE) is an uncommon, clinically distinct side effect of epidermal growth factor receptor (EGFR) inhibitors.

**Patient concerns:**

Unlike acneiform eruption, which arises from hair follicles mainly in the head and neck area, PDE starts from xerosis cutis, primarily in the lower extremities and is not associated with hair follicles. Herein, we report 3 cases of 3 patients who had received EGFR inhibitor and were hospitalized for PDE later. The cases were characterized by painful late-onset palpable purpura with identifiable bacterial pathogens.

**Diagnosis:**

The patients were diagnosed with characteristic clinical presentations, that is, late onset, PDE locations mainly in the lower extremities, nonfollicular centricity, and laboratory findings with identifiable bacterial pathogens.

**Interventions:**

Systemic antibiotics and intensive moisturizer application were prescribed.

**Outcomes:**

All the patients were successfully treated within 6 to 9 days without discontinuation of EGFR inhibitors.

**Conclusion:**

Systemic antibiotics, topical emollient, and skin barrier repair should be included in the treatment regimens for PDE.

## 1 Introduction

Epidermal growth factor receptor (EGFR) inhibitors block the signal transduction pathway to inhibit cancer cell proliferation and survival and are widely used to treat non-small cell lung cancer.^[1,2]^ Because of the distribution of EGFR, the most common side effects of EGFR inhibitors are cutaneous adverse events that are crucially dependent on EGFR signaling for normal function, and these adverse events affect 50% to 100% of patients, in a dose-dependent manner.^[3]^ Skin-related side effects can result in decreased quality of life and may require decrease in the dose of EGFR inhibitors or interruption or discontinuation of cancer treatment. Thus, proper management and prevention of possible skin-related side effects are important for continuing EGFR inhibitor treatment.^[4,5]^

The most common skin toxic side effect is acneiform eruption, and other common events include pruritus, xerosis, and paronychia.^[6]^ Less common adverse effects include mucositis, trichomegaly, and hypersensitivity. Purpuric drug eruption (PDE) has been reported as a rare complication.^[7]^ However, the prevalence of PDE may not be as rare as expected. Recently, we encountered 3 hospitalized PDE patients. They were treated successfully with both systemic antibiotics and intensive skin care, without a decrease or discontinuation of EGFR inhibitor. By sharing these cases, we can increase clinicians’ awareness of PDE to provide timely and appropriate treatment in order to improve patient quality of life and avoid interruption of treatment with EGFR inhibitors.

## 2 Case presentation

(Each of following 3 patients has provided informed consent for publication of the case.)

**Figure 1:**
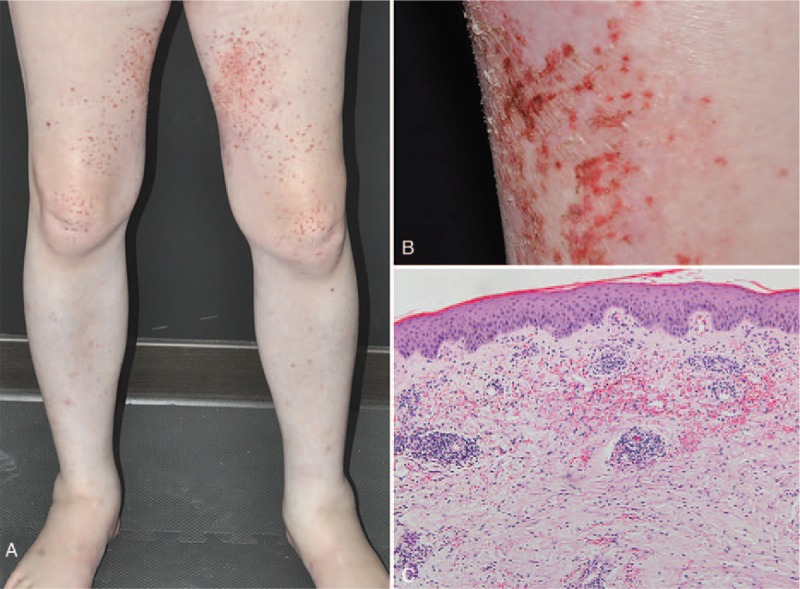
Case 1. (A) Multiple purpuric papules and pustule on erythematous to violaceous patches and erosions over his bilateral thighs and legs. (B) Xerosis skin over his thigh. (C) Histopathology showed telangiectasia, erythrocyte extravasation, and a perivascular inflammatory infiltration composed with neutrophils and lymphocytes in the superficial dermis without vasculitis. (100×; hematoxylin and eosin stain).

Case 1: A 61-year-old man had stage IB lung adenocarcinoma with EGFR mutation (+), exon 21 L858R, and received erlotinib 150 mg daily. Two and a half months after erlotinib was started, he initially experienced xerotic (dry) change over his bilateral lower extremities. Then, purpuric macules and papules were noted, and some papules turned into pustules days later. Clinical inspection showed multiple purpuric papules and pustules that were topped with crust, and erythematous-to-violaceous plaques and erosions over his bilateral thighs and legs (Fig. 1 A and B). Histopathology showed epidermal atrophy, parakeratosis, exudate with bacterial colonies on the surface, and neutrophil and lymphocyte inflammatory cell infiltration into the upper dermis (Fig. 1C). The dermis also showed telangiectatic changes with erythrocyte extravasation and fibrin deposition into the vascular wall of blood vessels (Fig. 1C). No fungus was found in the periodic acid-Schiff stain. The laboratory examination showed normal platelet counts, prothrombin time, and partial thromboplastin time. Pus and tissue cultures yielded oxacillin-susceptible *Staphylococcus aureus* (OSSA) and *Pseudomonas aeruginosa*. Systemic treatment with cefepime for 1 week and skin care with topical emollient were given. The patient continued erlotinib treatment during the treatment course, and the skin eruption subsided after 9 days of therapy. During the following 3 months, if he did not apply topical emollient, xerotic skin and subsequent tender pustules appeared within days. And this kind of attack was experienced 3 times.

Case 2: An 82-year-old woman was diagnosed with stage IV lung adenocarcinoma with an EGFR mutation (exon 21 L858R) and was treated with oral gefitinib 250 mg daily. After 3 months of gefitinib treatment, multiple erythematous and purpuric macules, papules, and stinging erosions appeared over her buttocks, bilateral inner thighs, knee fossae, and bilateral inner legs, especially at her bilateral inner thigh regions. No mucosal lesions were observed. Skin biopsy showed epidermal atrophy, necrosis, spongiosis, and marked neutrophilic infiltration and erythrocyte extravasation into the superficial dermis, with vasculitis changes of small blood vessels that showed fibrinoid deposition and neutrophils with predominant vascular infiltration. Suprabasal and subcorneal acantholysis were also noted. A direct immunofluorescence study showed negative results, and the laboratory tests, including platelet count and coagulation profiles, were within normal limits. Bacterial culture from the erosions yielded oxacillin-resistant *Staphylococcus aureus* (ORSA). Treatments with oral minocycline and potent topical corticosteroids (fluocinolone acetonide) and emollient were given without the discontinuation of gefitinib therapy. One week later, the skin eruption subsided with hyperpigmentation without recurrence during the following 6 months.

Case 3: A 63-year-old woman was diagnosed with stage IV lung adenocarcinoma with an EGFR mutation (+) (exon 21 L858R) and received erlotinib treatment 150 mg daily. Two and half months later, multiple severe painful and itchy discrete erythematous to purpuric papules, pustules, and crusted ulcers on her chest, abdomen, pubic area, back, and 4 limbs were noted. The skin biopsy revealed parakeratosis, basal cell vacuolization, perivascular lymphocytic, and neutrophilic infiltration, with erythrocyte extravasation into the superficial dermis and gram-positive cocci in small clusters that were compatible with the culture result. Amyloid deposition was noted at the papillary dermis. The periodic acid-Schiff stain showed negative results for fungus. Her platelet count and coagulation profiles were within normal limits, and the pus culture yielded OSSA. She received treatment with systemic cefazolin and topical petrolatum without discontinuation of erlotinib treatment. The skin eruption subsided after 6 days of treatment.

## 3 Discussion

PDE is clinically distinct from acneiform skin eruption. Although there is no large-scale epidemiologic study to explore the incidence of PDE, PDE seems not as rare as expected, according to our experiences. Among the skin toxicities that are associated with EGFRIs, acneiform eruption is the most common. The link between acneiform eruption and the development of PDE is not clear. The 3 patients presented here all had grade 2 acneiform eruptions on the face, chest, and back 10 to 21 days after starting EGFR inhibitor treatment, and all of the acneiform lesions subsided within 2 weeks of proper treatment (Table 1). The time frame of PDE is quite different from that of acneiform eruption. The median interval between the development of PDE and EGFR inhibitor commencement is 2.5 to 3 months in our patients and 3.5 months in 1 previous report.^[7]^ This is longer than that of acneiform eruption, of which the median time to onset ranges from 1 to 2 weeks,^[4,5]^ often reaching a maximum at 2 to 3 weeks following therapy initiation.^[3]^

**Table 1:**
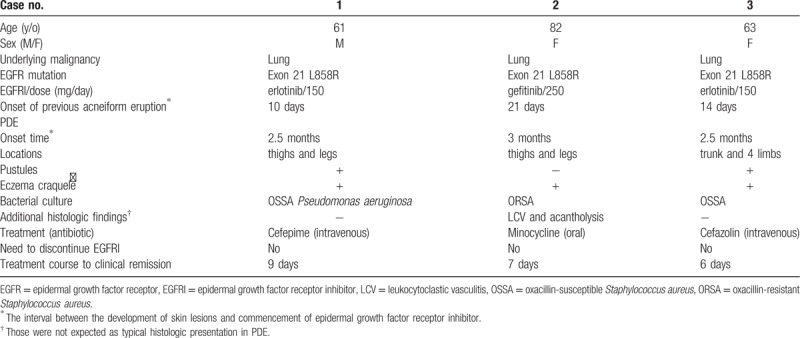
Summary of characteristics in these 3 purpuric drug eruption patients.

The cutaneous manifestations of PDE are multiple purpuric erythematous papules, which frequently present various sized pustules and can even become coalesced purpuric erosions. These lesions are not follicular centric while acneiform eruptions invariably arise from hair follicles. PDE shows a predominant distribution in the lower extremities, and other less frequent locations include the upper extremities and trunk. The face is usually spared, while acneiform eruption invariably involves seborrheic (oily) area, including the face, scalp, and chest.^[7,8]^

The pathogenesis of PDE involves a mixture of different pathways. Skin barrier and bacteria may play an important role, and the bacterial cultures from our 3 hospitalized patients all yielded *S aureus*, and one also showed *P aeruginosa*. The result is consistent with previous studies, which revealed *S aureus* was the most common bacterial pathogen in patients treated with EGFR inhibitors, and the second was *P aeruginosa*.^[7,9]^ Treatment with systemic antibiotics is effective. On the contrary, bacterial cultures of acneiform eruption lesions usually show negative results, except in cases of secondary superinfection.^[8]^

Furthermore, we observed PDE lesions invariably started from the xerosis skin. Eczema craquelé (asteatotic dermatitis) appears initially (Fig. 1B); then, purpuric eruptions appear in the dry cracking skin and finally turn into tender and painful pustules rapidly within several days when proper treatment is not given.^[10]^ Xerosis is a common cutaneous adverse effect of EGFR inhibitors and appears relatively late, about 1 to 2 months after the start of EGFR inhibitor.^[11]^ Since there is a close association between skin xerosis and PDE, proper skin care with emollient and skin barrier repair should be crucial parts of the treatment for PDE.

The histopathologic findings of PDE showed a subcorneal pustule without folliculitis in a pustule lesion. Epidermal atrophy, parakeratosis, focal hydropic degeneration, or spongiosis could be found in some of the reported cases. The superficial dermis in a purpuric papule commonly shows perivascular neutrophilic and lymphocytic infiltration and telangiectatic changes with abundant erythrocyte extravasations (Fig. 1C). Some cases also revealed leukocytoclastic vasculitis changes; however, these changes may be secondary to ulcer-induced inflammation.^[7,10]^ In Case 2, there were both suprabasal and subcorneal acantholysis (loss of cohesion between keratinocytes). Acantholysis can appear in autoimmune blister disease, such as pemphigus, but can be incidentally found in many inflammatory dermatoses, such as staphylococcal scalded skin syndrome. Acantholysis in PDE is an uncommon histological presentation that has only been reported in 3 cases.^[12,13]^ In our case, the direct immunofluorescence study showed negative results; thus, autoimmune bullous diseases were less likely. The mechanism may involve *S aureus* exfoliative toxin A targeting desmoglein 1, which results in subcorneal acantholysis.^[14]^ Another possible hypothesis is that activated neutrophils that are induced by EGFR inhibitors may release proteases that contribute to further tissue destruction, with the loss of intercellular attachments in the epidermis, basal keratinocyte degeneration, and destruction of the basement membrane.^[15]^ Amyloid deposition in papillary dermis was found incidentally in case 3, and there was no related clinical change.

EGFR is expressed on basal epidermal keratinocytes, the outer root sheath cells of hair follicles, sebaceous and eccrine sweat gland cells, some endothelial cells, smooth muscle cells of dermal vessels, and various cancer cells.^[2]^ Disruption of the normal EGFR pathway of basal keratinocytes can give rise to growth arrest and premature differentiation, leading to impaired stratum corneum, interference of sebaceous gland function, and reduced expression of major components of cornified cell envelopes, which results in loss of the water-retaining function of the epidermis, and then xerosis skin develops.^[15]^ Additionally, the release of inflammatory cell chemoattractants may recruit leukocytes that release enzymes, resulting in apoptosis and tissue damage with subsequent apoptotic keratinocytes, vascular dilation, and increased permeability.^[15]^ The purpuric change may be involved in a similar mechanism. The EGFR on endothelial cells and dermal vessel smooth muscle cells and EGFR inhibitors can result in inflammation of the endothelium, loss of vessel wall structural support, increased permeability, and red blood cell extravasation. In addition, xerosis with disturbed barrier function also increases susceptibility to injuries and facilitates microbial invasion. In human skin, antimicrobial peptides such as human β-defensins (hBDs) serve as the first line of defense against infections by pathogenic microorganisms. EGFR inhibitor could dampen the innate immune system and suppress hBD2 expression,^[7,16]^ which allows bacterial infection and further results in aggravating tissue inflammation, recruiting neutrophils, and the subsequent formation of pustules.

According to the speculated mechanisms described as the above, factors including impaired barrier function, perivascular inflammation, and pathogen invasion can result in a clinical presentation that includes xerosis skin, purpuric change, and pustule formation. Therefore, we recommend routine microorganism surveys for purpuric and pustules lesions, that is, for doctors to treat such findings with definitive antibiotic therapy, based on the culture report. Before the final culture report, an antibiotic should be selected to treat OSSA, ORSA, and *P aeruginosa*. Our experiences strongly suggest that intensive skin care is as important as antibiotic treatment for controlling and reestablishing normal skin components and preventing microorganism invasion, which avoid further inflammation and pustule formation. Besides, the moisture contents of the horny layer and cutaneous sebum levels increased after moisturizer application, which is effective against dry skin and skin toxicities due to EGFR inhibitors.^[17,18]^ All of the patients in our case report were successfully treated with systemic antibiotics with intensive moisturizer, without decreasing or discontinuing EGFR inhibitors. The median clinical treatment course in our 3 cases was 6 to 9 days.

In summary, we share 3 cases of EGFR-induced PDE who were successfully treated with systemic antibiotics for 6 to 9 days with intensive moisturizer, without decreasing or discontinuing EGFR inhibitors. The median clinical treatment course in our 3 cases was 6 to 9 days. The clinical manifestations of PDE differ from those of acneiform eruptions with regard to late onset, location mainly at the lower extremities, nonfollicular centricity, and identifiable bacterial pathogens. It should be emphasized the importance of timely treatment with systemic antibiotics, topical application moisturizer, and avoiding discontinuation of EGFR inhibitors to achieve better clinical outcomes of cancer treatment.

## Author contributions

**Conceptualization:** Szu-Yun Fang, Kai-Che Wei.

**Supervision:** Yi-Shan Liu, Kai-Che Wei.

**Writing – original draft:** Szu-Yun Fang, Kai-Che Wei.

**Writing – review & editing:** Chieh-Shan Wu.
